# Prolonged Mechanical Ventilation Alters the Expression Pattern of Angio-neogenetic Factors in a Pre-Clinical Rat Model

**DOI:** 10.1371/journal.pone.0070524

**Published:** 2013-08-08

**Authors:** Christian S. Bruells, Karen Maes, Rolf Rossaint, Debby Thomas, Nele Cielen, Christian Bleilevens, Ingmar Bergs, Ursina Loetscher, Agnes Dreier, Ghislaine Gayan-Ramirez, Brad J. Behnke, Joachim Weis

**Affiliations:** 1 Department of Anesthesiology, University Hospital of the RWTH Aachen, Aachen, Germany; 2 Department of Surgical Intensive and Intermediate Care, University Hospital of the RWTH Aachen, Aachen, Germany; 3 Laboratory of Pneumology, Katholieke Universiteit Leuven, Leuven, Belgium; 4 Institute of Neuropathology and JARA – Translational Brain Medicine, Aachen University Hospital of the RWTH Aachen, Aachen, Germany; 5 Institute of Applied Physiology and Kinesiology, College of Health and Human Performance, University of Florida, Gainesville, Florida, United States of America; National Institutes of Health, United States of America

## Abstract

**Objective:**

Mechanical ventilation (MV) is a life saving intervention for patients with respiratory failure. Even after 6 hours of MV, diaphragm atrophy and dysfunction (collectively referred to as ventilator-induced diaphragmatic dysfunction, VIDD) occurs in concert with a blunted blood flow and oxygen delivery. The regulation of hypoxia sensitive factors (i.e. hypoxia inducible factor 1α, 2α (HIF-1α,–2α), vascular endothelial growth factor (VEGF)) and angio-neogenetic factors (angiopoietin 1–3, Ang) might contribute to reactive and compensatory alterations in diaphragm muscle.

**Methods:**

Male Wistar rats (n = 8) were ventilated for 24 hours or directly sacrificed (n = 8), diaphragm and mixed gastrocnemius muscle tissue was removed. Quantitative real time PCR and western blot analyses were performed to detect changes in angio-neogenetic factors and inflammatory markers. Tissues were stained using Isolectin (IB 4) to determine capillarity and calculate the capillary/fiber ratio.

**Results:**

MV resulted in up-regulation of Ang 2 and HIF-1α mRNA in both diaphragm and gastrocnemius, while VEGF mRNA was down-regulated in both tissues. HIF-2α mRNA was reduced in both tissues, while GLUT 4 mRNA was increased in gastrocnemius and reduced in diaphragm samples. Protein levels of VEGF, HIF-1α, -2α and 4 did not change significantly. Additionally, inflammatory cytokine mRNA (Interleukin (IL)-6, IL-1β and TNF α) were elevated in diaphragm tissue.

**Conclusion:**

The results demonstrate that 24 hrs of MV and the associated limb disuse induce an up-regulation of angio-neogenetic factors that are connected to HIF-1α. Changes in HIF-1α expression may be due to several interactions occurring during MV.

## Introduction

Mechanical ventilation (MV) is a life-saving intervention in patients with respiratory insufficiency. Despite its benefits, prolonged MV has been shown to result in contractile dysfunction and atrophy in diaphragm tissue, a condition collectively termed ventilator-induced diaphragm dysfunction (VIDD) [1], [2]. Additionally, recent data indicate a severe reduction in diaphragm blood flow and oxygen delivery to the diaphragm after 6 hours of MV [3], which was not present in other skeletal muscle subject to identical temporal and anesthetic parameters. Indeed, in contrast to the diaphragm, 12 hours of MV does not result in atrophy, loss of specific force generation or reduced blood flow [4] [3] of limb muscle.

Several factors regulate the tissue response to altered oxygen supply and/or changes in blood flow in order to initiate changes in blood vessel architecture. For example, the hypoxia inducible factor (HIF)-1α protein is a heterodimer consisting of O_2_ sensitive subunits and is expressed in all mammalian cell types [5], [6]. In well oxygenated tissue, HIF-1α is regulated on the protein level in an oxygen sensitive manner, being hydroxylated and degraded by the proteasome [5]. HIF-1α is also regulated by inflammatory cytokines such as interleukin (IL)-1β [7], IL-6 [8] and tumor necrosis factor (TNF)-α via the nuclear factor-κB (Nf-κB) pathway [9]. HIF-1α acts as a transcription factor for a variety of genes, including vascular endothelial growth factor (VEGF), a powerful modulator of vessel neogenesis in response to hypoxia [10]. In concert with VEGF, vasculogenesis is regulated, in part, by the angiopoietin (Ang) family transcription factors that are expressed in different isoforms (Ang 1–4) [11], [12]. While the binding of Ang 1 leads to stabilization of the microvasculature, vessel structure is destabilized prior to remodeling by the binding of Ang 2. Capillary sprouting has been described in the presence of VEGF, while in its absence a capillary regression is initiated [11].

Very little is known about the changes of angio-neogenetic factors during MV, and potential mechanisms. Previously, a two-fold reduction of VEGF mRNA expression was reported in the diaphragm after 18 hours of MV in a rat model [13]. However, with 90 min of MV VEGF mRNA expression was elevated in human patients [14], although the effect of longer-term ventilation on other growth factors is unknown. In the current study, we hypothesized that 24 hours of MV would result in a significant increase in angio-neogenetic factors and, subsequently, diaphragm capillary density as a compensatory response to the reduced oxygenation of the diaphragm with prolonged MV [3].

To test our hypothesis, we used an established rat model of MV applied for 24 hours to investigate the angio-neogenetic response in diaphragm and mixed gastrocnemius muscle tissue (also subject to disuse during this time frame) for mRNA expression and protein concentration of angio-neogenetic and hypoxia sensitive factors. Furthermore, we combined these potential changes in signaling mechanisms with histological evaluations of capillary/fiber ratio.

## Methods

### Experimental design

To test the hypothesis that MV changes the expression pattern of angio-neogenetic factors, adult male Wistar rats (n = 16; 400 g) were randomly assigned to one of two groups: 1) Control, acutely anesthetized and sacrificed (n = 8), and 2) mechanically ventilated for 24 h (MV; n = 8). The work was approved by the animal care committee of Katholieke Universiteit Leuven (P071/2012). All procedures were performed under sodium pentobarbital anesthesia as this general anesthetic does not affect muscle force [15] or alter skeletal muscle blood flow [3] with prolonged MV.

### Ventilation protocol

Rats were ventilated using an established ventilation protocol [2] or were acutely anesthetized and euthanized. During MV, anesthesia with sodium pentobarbital was maintained using the central venous line after an initial intraperitoneal injection (50 mg/kg body weight). Throughout MV, animals received 0.9% saline solution through the jugular vein at a rate of 0.6 ml/h to maintain hydration and supplementary doses of the anesthetic sodium pentobarbital at 1.2 mg/100 g body weight/h. Arterial blood pressure was recorded (BP2, Columbus instruments, Columbus, OH, USA) and blood collected for measurement of arterial PaO_2_ and PaCO_2_ via a catheter placed in the carotid artery. All animals demonstrated a mean arterial pressure above 90 mmHg during the experiments. As part of routine surgical care, the rats were rotated, the eyes were lubricated and the bladder expressed. Temperature was maintained at 37°C using a heating blanket and measured via a rectal temperature probe. At the end of the experiments, the diaphragm and mixed portion of the gastrocnemius muscle were removed and stored at −80°C for histological and biochemical analysis.

### Histology

Tissue was embedded in OCT compound (Tissue Tek, The Netherlands) and frozen at −80°C in butene for cutting and mounting on slides. Muscle tissue sections (7 μm) were stained using antibodies against Isolectin GS-IB_4_ from *Griffonia simplicifolia*, biotin-XX Conjugate (Life Technologies Corp., Carlsbad, CA, USA), a 114 kDa glycoprotein and part of a family of five tetrameric type I isolectins, at a concentration of 1∶2000 to stain endothelial cells. Capillaries adjacent to 100 diaphragm and mixed gastrocnemius muscle fibers per animals were counted. As a potential marker of tissue hypoxia, muscle tissue was stained using GLUT 1 antibody (Ab 15309, Abcam, Cambridge, UK), whose expression has been shown to be significantly related to binding of the bioreductive drug hypoxia marker pimonidazol [16]. As secondary antibody, Rhodamine red (Invitrogen, Frankfurt, Germany) was used for both staining protocols. Pictures were taken using Fluorescence microscopy (Zeiss Axiovision, Jena, Germany) at 400-fold magnification.

### Messenger RNA quantification by real-time reverse-transcription polymerase chain reaction (RT-PCR)

Diaphragm samples and mixed gastrocnemius muscle tissue samples (30 mg) were snap frozen in liquid nitrogen and stored at −80°C. Total mRNA was extracted using the guanidiumthiocyanate selective silica-gel membrane-binding method (Rneasy Fibrous Tissue Mini Kit #74704, Qiagen, Valencia, CA, USA), based on manufacturer’s instructions. Two-step real-time RT-PCR was performed for the relative quantification of mRNA (QuantiTect reverse transcription kit #205311, QuantiTect SYBR Green kit #204054, Qiagen; StepOne real-time PCR system, Applied Biosystems, Foster City, CA, USA). PCR threshold cycles (CT) for *Ang 1,2,3*, *HIF-1*α, *HIF-2*α and *GLUT 4* as a well as *IL-6*, *TNF-*α and *IL-1β*were corrected by the CT value of the endogenous reference Hypoxanthinephophoribosyl-transferase (HPRT) (Relative quantity [RQ]). RQ corresponds to the fold change between the sample from MV animals and the pool from control animals acutely anesthetized, seperately for each muscle (RQ  =  2^ΔCTsample–ΔCTcontrol^)).

### Western blot

A maximum of 30 mg of diaphragm and mixed gastrocnemius tissue was homogenized on ice in 800 µl lysis buffer containing 150 mM sodium chloride, 1.0% NP-40, 0.1% sodium dodecyl sulfate (SDS), 1% sodium deooxycholate, 50 mM Tris-HCl (pH 7.6; all from Sigma-Aldrich, Germany) and protease inhibitor cocktail tablets (Roche Diagnostics, Mannheim, Germany), using 2 ml Potter-S homogenization cylinders (Sartorius; Goettingen, Germany). Homogenates were centrifuged through Qiagen-shredder columns (Qiagen, Hilden, Germany) at 2000 rpm for 2 min and the supernatant was used for the determination of protein concentrations, using a DC-Protein Assay Kit (Bio-Rad Laboratories, München, Germany). Samples were boiled for 5 min after addition of Laemmli-buffer (312.5 mM Tris HCl, pH 6.8, 10% SDS, 50% Glycerin, 10% β-Mercaptoethanol, less than 5 mg Bromphenol-blue; all from Sigma-Aldrich). An equal amount of 20 µg of each sample was separated by 10% SDS-Page and transferred onto a PVDF membrane (Bio-Rad Laboratories). After the semi-dry blotting procedure (60 min, 25 V), the membrane was incubated for 1 h at room temperature (RT) in 5% BSA blocking-solution (Albumin fraction V; ROTH, Karlsruhe, Germany), followed by overnight incubation on a shaker at 4°C with specific antibodies for VEGF (sc-57496; Santa Cruz biotechnology, California, USA), HIF-1α (07-628, Millipore, Billerika, MA, USA,), HIF-2α (NB100-132) and GLUT 4 (NB 100-79958, both Novus Biologicals, Littleton, CO, USA), IκBa (#4812, Cell Signaling Technology, Danvers MA, USA) and p65 subunit of NfkB (#4764, Cell Signaling Technology, Danvers MA, USA) and GAPDH as a loading control (#5174; Cell Signaling Technology, Danvers MA, USA). Overnight incubation was followed by repeated washing steps (3×5 min in TBS buffer containing 1% Tween20; Sigma Aldrich), then the membrane was incubated for 1 h at RT on a shaker with horseradish-peroxidase conjugated goat anti-rabbit antibody (#7074; Cell Signaling Technology, Danvers MA, USA). The final reaction was visualized using enhanced chemiluminescence (WEST-ZOL Plus Western Blot Detection System; iNtRON Biotechnology, Korea) and a detection system (BioDocAnalyze Live; Biometra, Goettingen, Germany). Images were taken and densitometrically analyzed using ImageJ (v1.46k; National Institute of Health, Bethesda, MA, USA). HEY cells (ovarian cancer cell line, kind gift from Prof Meinhold – Heerlein, Aachen, [17]), which had been incubated in an hypoxic chamber for 96 h were used as positive controls for occurrence of hypoxia related proteins (HIF-1α, 2α and GLUT 4) [17]. For Western blot pictures indicating proper antibody binding (see Figure S2).

### Statistical analysis

PCR data were calculated with the control pool set as 1 and changes in expression levels were calculated using a students t-test. To detect differences in capillary/fiber ratio and differences in protein expression levels, an unpaired t-test was used. Significance was defined at p<0.05. Data are shown as means ± SD unless otherwise specified.

## Results

### Systemic and biological responses

Mean arterial pressure (140±23 mmHg), Pa_O2_ (127.8±27.1 mmHg) and Pa_CO2_ (23±6.4 mmHg) were maintained relatively constant throughout the 24 hour MV period.

### MV and mRNA expression of angio-neogenetic factors

**Figure 1:**
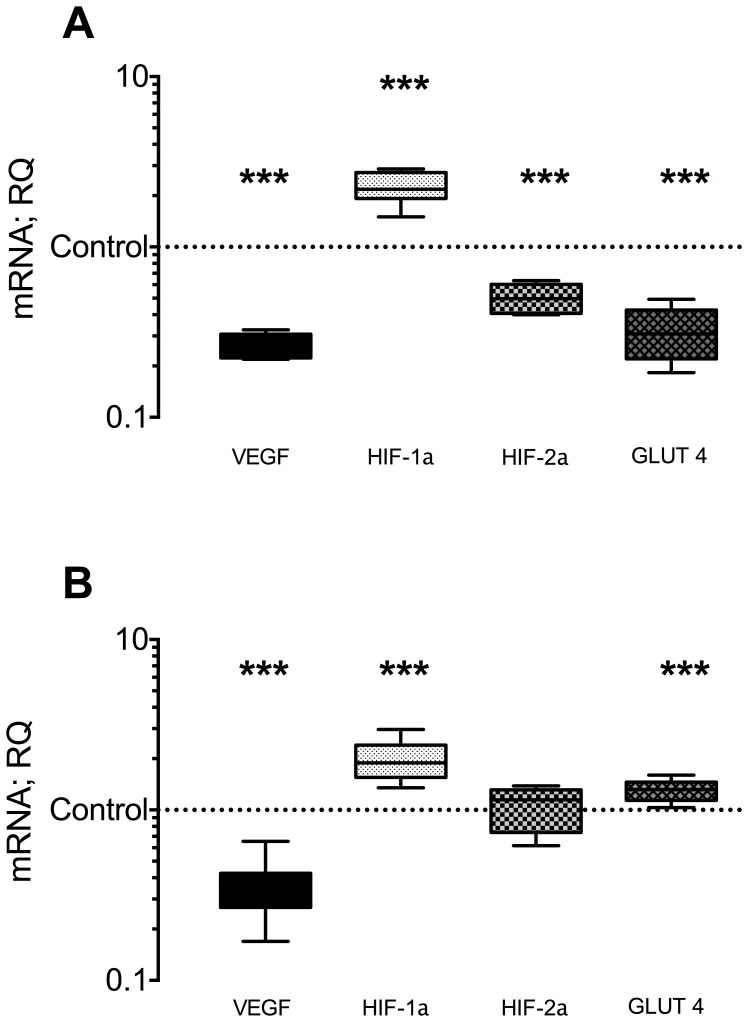
mRNA relative expression of angio-neogenetic factors in ventilated animals compared to unventilated controls. Panel (A) shows changes in diaphragm muscle tissue, panel (B) changes in gastrocnemius muscle tissue. ** indicate p<0.001, *** p<0.0001. Ang = Angiopoietin, RQ relative quantity. Further explanation see text. The horizontal line, box, and whiskers of each box plot represent the median, the interquartile range, and the upper and lower range of the data, respectively. n = 8/per group.

MV induced a significant up-regulation of Ang 2 mRNA in diaphragm and gastrocnemius tissue, while Ang 1 and 3 mRNA were down-regulated only in gastrocnemius tissue (see Figure 1, and Table 1). Ang 1 and Ang 3 mRNA were unchanged in diaphragmatic tissue.

**Table 1:** p-values and fold-changes of mRNA expression in diaphragm and mixed gastrocnemius tissue of MV compared to control.

Diaphragm	Fold change	p-value
Angiopoietin 1	0.85	0.19
Angiopoietin 2	2.046	0.005*
Angiopoietin 3	0.98	0.95
Vascular endothelial growth factor	0.25	<0.001*
Hypoxia inducible factor 1α	2.25	<0.001*
Hypoxia inducible factor 2α	0.50	<0.001*
Glucose transporter 4	0.31	<0.001*
Tumor necrosis factor α	1.97	<0.01*
Interleukin 6	1.85	<0.05*
Interleukin 1β	1.56	0.23

Mixed Gastrocnemius	Fold change	p-value
Angiopoietin 1	0.61	<0.001*
Angiopoietin 2	2.45	0.004*
Angiopoietin 3	0.38	<0.001*
Vascular endothelial growth factor	0.37	<0.001*
Hypoxia inducible factor 1α	1.97	0.001*
Hypoxia inducible factor 2α	1.052	0.64
Glucose transporter 4	1.31	0.002*
Tumor necrosis factor α	0.51	<0.01*
Interleukin 6	2.67	<0.05*
Interleukin 1β	1.6	<0.05*

* indicates significant change versus control.

### MV and VEGF and HIF-1α, HIF-2α and GLUT 4 mRNA expression

**Figure 2:**
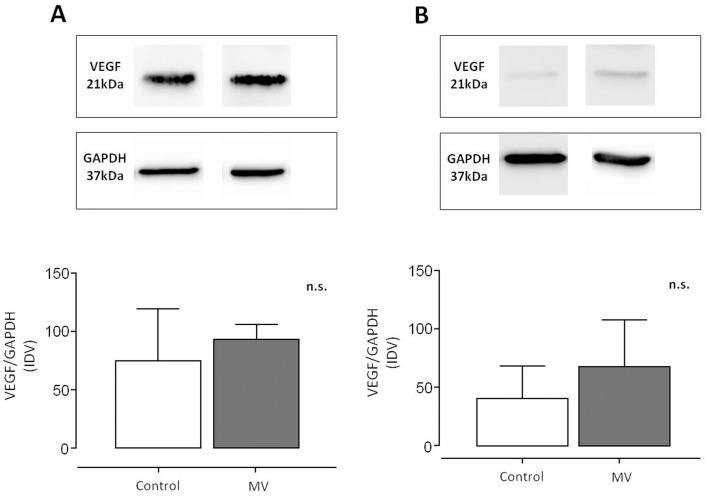
mRNA relative expression of hypoxia regulated transcription factors in ventilated animals compared to controls. Panel (A) shows changes in diaphragm muscle tissue, panel (B) changes in gastrocnemius muscle tissue. ** indicate p<0.001, *** p<0.0001. HIF = hypoxia inducible factor, VEGF = vascular endothelial growth The horizontal line, box, and whiskers of each box plot represent the median, the interquartile range, and the upper and lower range of the data, respectively. n = 8/group.

In order to investigate the result of MV on hypoxia-induced transcription factors, we measured VEGF and HIF-1α mRNA expression. A significant up-regulation of HIF-1α mRNA was found in both diaphragm and gastrocnemius compared to control after MV (see Figure 2 and Table 1). Conversely, a significant down-regulation of VEGF mRNA was measured in both diaphragm and gastrocnemius compared to control (see Figure 2 and Table 1). In contrast to HIF-1α, HIF-2α mRNA expression was significantly reduced following MV in diaphragm tissue (see Figure 2, Table 1). Expression of GLUT 4 mRNA was significantly diminished in diaphragm tissue, while in gastrocnemius muscle, GLUT 4 was significantly up-regulated compared to controls (see Figure 2 and Table 1).

### 24 hours of MV does not increase protein abundance of hypoxia related proteins

**Figure 3:**
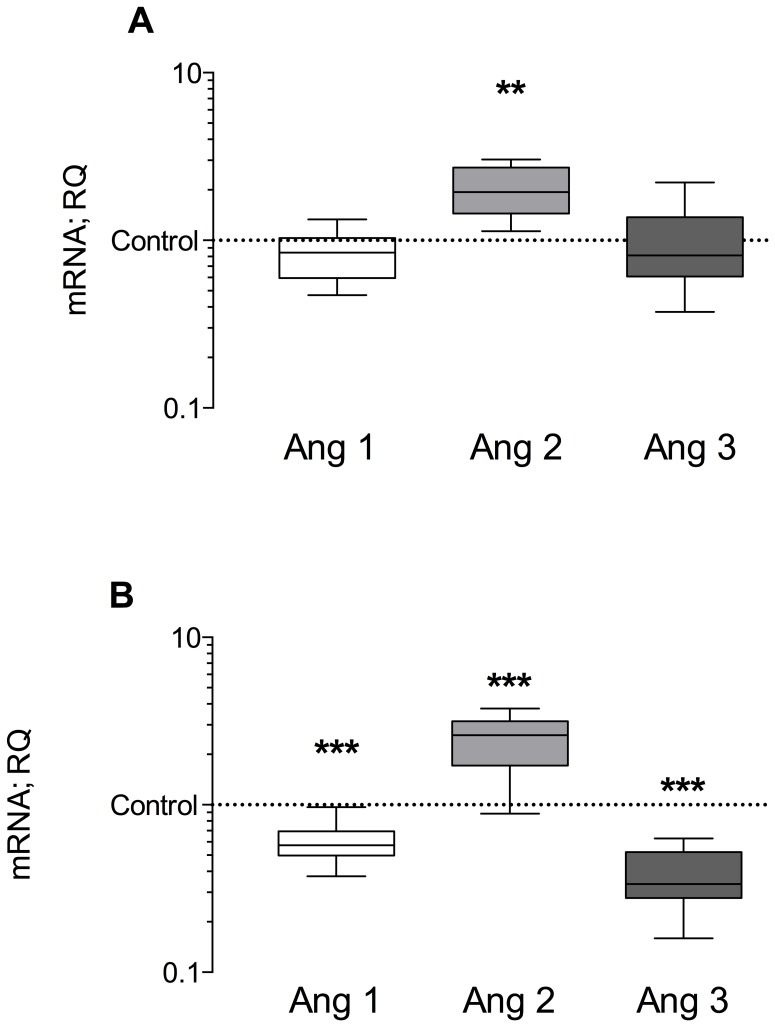
VEGF protein levels in ventilated animals compared to controls. Panel (A) shows changes in diaphragm muscle tissue, panel (B) changes in gastrocnemius muscle tissue. VEGF levels are maintained in the diaphragm during 24 hours of Mechanical ventilation/disuse, although there is a strong trend in gastrocnemius for up-regulation (p = 0.07). Representative western blot bands are depicted above the graph. CON = Controls n = 7, MV = Mechanical ventilation, Diaphragm n = 8, MV Gastroc n = 6. Exposition times: VEGF 30 sec each; GAPDH 2 sec.

Although mRNA expression of hypoxic sensitive factors in both diaphragm and gastrocnemius changed during MV, 24 hours of MV did not change VEGF protein concentrations in diaphragm (see Figure 3) or gastrocnemius samples. Interestingly, in the diaphragm, no increase in protein abundance of HIF-1α, HIF-2α and GLUT 4 could be measured following MV (see Figure S2). In the gastrocnemius, no significant changes in HIF-1α or GLUT 4 protein levels were observed compared to controls (see Figure S1).

### 24 hours of MV does not alter GLUT 1 mRNA expression

As a potential marker of tissue hypoxia, GLUT 1 abundance was assessed using immunohistochemical staining. In gastrocnemius and diaphragm muscle, GLUT 1 protein expression was unchanged following 24 hours of MV (see Figures S3 and S4).

### MV induces an increase in inflammatory signaling in the diaphragm

**Figure 4:**
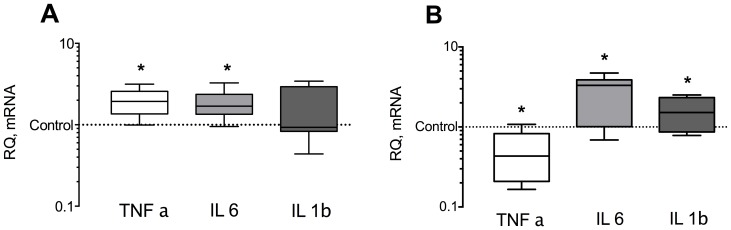
mRNA relative expression of inflammatory cytokines in ventilated animals compared to controls. Panel (A) shows changes in diaphragm muscle tissue, panel (B) changes in gastrocnemius muscle tissue. * indicate p<0.05. IL  =  Interleukin TNF = Tumor necrosis factor. RQ relative quantity. Further explanation see text. The horizontal line, box, and whiskers of each box plot represent the median, the interquartile range, and the upper and lower range of the data, respectively. n = 8/per group.

**Figure 5:**
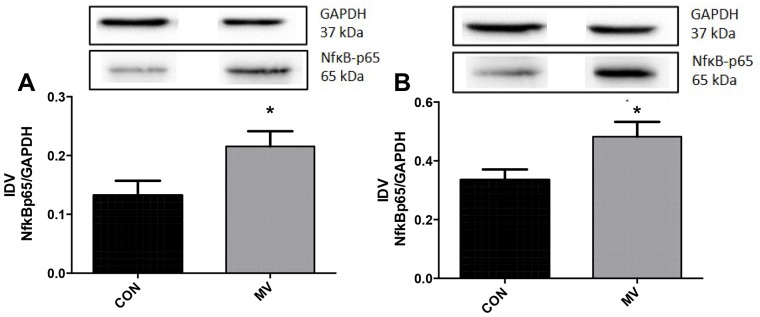
p65 subunit of Nuclear factor kappa B protein levels in ventilated animals compared to controls. Panel (A) shows changes in diaphragm muscle tissue, panel (B) changes in gastrocnemius muscle tissue. During MV, p65 is significantly increased in both tissues compared to unventilated controls. Representative western blot bands are depicted above the graph. CON = Controls, MV = Mechanical ventilation. Controls n = 7, MV Diaphragm n = 8, MV Gastroc n = 6. Exposition times: Diaphragm 2 Min/GAPDH 6 sec, Gastroc 3 Min/GAPDH 7 sec.

In diaphragm tissue, MV led to a significant increase in the expression of TNF-α and IL-6 mRNA, while in gastrocnemius muscle tissue, IL-6 and IL-1β mRNA were significantly up-regulated and TNF-α mRNA was down-regulated (see Figure 4, and Table 1). To determine the upstream inflammatory signaling mechanism, we measured the p65 subunit of NfκB and the inhibitory protein IkappaB-α (IκBα) which otherwise binds NF-κB heterodimers and maintains it inactive in the cytosol [7], [18]. The protein concentration of p65 was significantly elevated in diaphragm and gastrocnemius tissue (see Figure 5), while IκB-α protein abundance did not change significantly (see Figure S5).

### 24 hours of MV does not affect capillarity in the diaphragm

**Figure 6:**
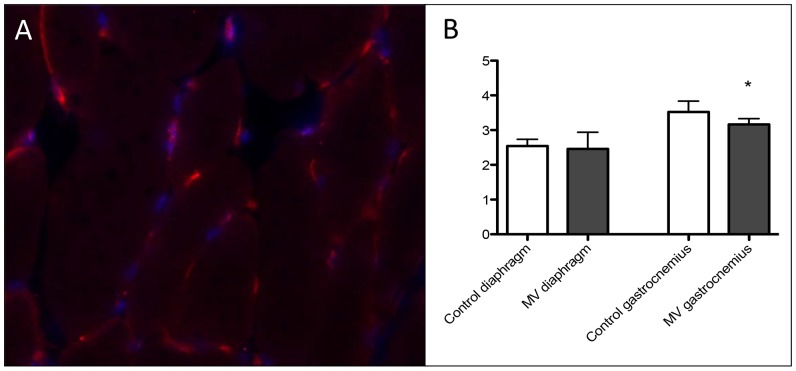
Changes in capillary/ fiber ratio in both tissues. Panel (A) shows an example of the staining in diaphragmatic tissue, Panel (B) depicts the capillary/fiber ratio in both tissues. Red dots in the picture indicate capillaries, while blue ones are DAPI stained nuclei. n = 8/group, control diaphragm n = 7; * p<0.05.

In the diaphragm, the number of capillaries per muscle fiber did not change significantly after MV (MV 2.45±0.5 vs. control 2.54±0.2 capillaries per fiber). In gastrocnemius, the capillary per fiber ratio was significantly reduced (MV 3.16±0.2 vs. control 3.47±0.3 capillaries per fiber, p = 0.01 (see Figure 6).

## Discussion

### Overview of principal findings

Twenty-four hours of MV altered expression of angio-neogenetic factors in the diaphragm. Surprisingly, similar adaptations were observed in the mixed gastrocnemius muscle. Based on our findings, these changes do not appear to be a result of tissue hypoxia, although this possibility cannot be discounted as diaphragm tissue oxygenation was not directly measured. Furthermore, our findings reveal an increase in inflammatory markers in both gastrocnemius and diaphragm, which may influence the regulation of angio-neogenetic factors. A detailed discussion of these results follows.

### Mechanical Ventilation, Angiopoientins, and VEGF

Angiopoietins act in concert with HIF-1α and VEGF to react to exercise, disuse, or blood flow restriction and induce capillary sprouting in the presence of VEGF [19] or rarefaction in its absence [11]. Ang1 mRNA expression was unchanged in the diaphragm and decreased in the gastrocnemius in our study, whereas Ang 2 mRNA expression was increased in both muscles. Importantly, increases in Ang 2 have been reported in limb ischemia [20] and activation of Ang 2 has been linked to HIF-1α induction [21], and prostaglandin expression [21]. The elevated HIF-1α mRNA levels, as observed in both gastrocnemius and diaphragm muscles (Figure 2), may have induced Ang 2 expression. This may lead to vessel sprouting [11] in regions with lowered blood flow and oxygen delivery, such as in the diaphragm during mechanical ventilation [3]. With respect to potential signaling from hypoxia, our findings of no significant change in Ang 3 mRNA in the diaphragm with MV are not consistent with the findings of Abdulmalek and colleagues [12], who demonstrated enhanced diaphragm Ang 3 mRNA, unaltered Ang 1 mRNA and reduced Ang 2 mRNA expression after 12 hours of hypoxia (FiO_2_ 0.09–0.1). This discrepancy may be related to 1) hypoxia during MV is not present, and/or 2) the animals in the study of Abdulmalek and colleagues were not subjected to MV and likely had an enhanced diaphragm contractile activity with hypoxic ventilation whereas animals in the current study had a completely quiescent diaphragm with MV.

Contrary to our hypothesis, there was a down-regulation of VEGF in the diaphragm with MV. This is in agreement with previous reports showing a comparable decrease in VEGF mRNA in the diaphragm after 18 hours of MV [22] and in the gastrocnemius after 12 hours of disuse [23]. In the present study, VEGF protein levels were, however, maintained despite decreased mRNA levels. This discrepancy between mRNA and protein levels has been reported before for hind limb muscle [24] and has been interpreted as regulatory effect at the translational level. Given alterations in blood flow could affect VEGF expression [25] the severe reduction in blood flow to the diaphragm with MV [3] could be a signaling mechanism for the reduced VEGF expression observed herein. Although during 6 h of disuse, the blood flow to the gastrocnemius is preserved [3], there are no data available on blood flow over 24 h of disuse in limb muscle, and a reduction in flow over this time frame to limb muscle is possible.

### Changes in HIF-1α expression with MV

Previous work has demonstrated a severe O_2_ delivery-to-demand mismatch within the diaphragm after prolonged MV, limiting the ability to increase O_2_ uptake during contractions [3], which suggests the potential for diaphragm tissue hypoxia with prolonged MV. Indeed, HIF-1α a key regulator of cell metabolism with a high sensitivity for intracellular oxygen levels, mRNA was elevated after MV in our study (Figure 2). However, we could not detect any HIF-1α protein in our diaphragm samples that were in line with the associated increase in mRNA expression. In addition to hypoxia, HIF-1α activity is dependent on various mechanisms and pathways that may interfere with its transcription or stability of the protein, including inflammation or reactive oxygen species [6].

To test the hypothesis, that MV-induced alterations of HIF-1αmight be due to tissue hypoxia, we measured factors that are known to increase in response to tissue hypoxia, such as HIF-2α, GLUT 4 and GLUT 1. HIF-2α alterations during limb muscle hypoxia are reported to result in increased capillary density [26]. We measured HIF-2α expression in diaphragm and gastrocnemius, and detected a decrease in HIF-2α mRNA in diaphragm tissue. We detected an increase in GLUT 4 mRNA in gastrocnemius tissue, which could be an initial indicator of chronic hypoxia after 24 hours of disuse, due to the induction of GLUT 4 in anaerobic metabolism [27]. Otherwise, our findings agree with Henriksen and colleagues, who demonstrated increased GLUT 4 mRNA transcription as response to disuse (and not hypoxia) after 3 days of hind limb suspension in soleus muscle [28]. Therefore, it seems reasonable that GLUT 4 changes in the gastrocnemius in our experiments are mainly due to disuse and not hypoxia. Furthermore, the reduction in GLUT 4 mRNA in diaphragm may be interpreted as response to changes in insulin-like growth factor (IGF) receptor I expression during MV [29]. Indeed, 24 hours of mechanical ventilation reduced levels of IGF 1 mRNA, which is tightly linked to GLUT 4 transcription [30]. Furthermore, GLUT 1 expression in muscle tissue, which is activated comparably to pimonidazole, a sensitive indicator of hypoxia [16], was not changed significantly compared to controls. In summary, we could not detect an increase in three oxygen dependent factors, which respond to tissue hypoxia. Therefore, in the time-frame of MV utilized herein it is unlikely that hypoxia induced the changes in HIF-1α mRNA observed (Figure 2); however, without a direct measure of intracellular O_2_ concentration in the diaphragm with prolonged MV this possibility cannot be definitively excluded.

### Inflammatory response due to MV

Previous work has shown that HIF-1α can be induced by inflammatory cytokines via the Nf-κB pathway [7], [9]. Earlier work in diaphragm revealed an increase in inflammatory signaling with elevation of IL-6 and IL-1β ανδKX, the rodent analogue of IL-8, after 8 h of MV [31]. These findings are congruent with our data showing elevated IL-6, Il-1β and TNF-α mRNA in diaphragm tissue after 24 hours of MV (Figure 4). Inflammatory cytokines are able to activate the Nf-κB pathway, known to be up-regulated during hind limb disuse atrophy [32] and are associated with VIDD [18]. Our findings reveal an activation of NF-κB and downstream cytokines in both diaphragm and gastrocnemius tissue, possibly contributing to changes of HIF-1α. Therefore, our findings in diaphragm tissue of increased HIF-1α mRNA levels compared to non-ventilated controls may be partially due to the inflammatory response of unloading skeletal muscle.

### Changes in capillary to fiber ratio

Twenty-four hours of mechanical ventilation did not result in any change in the capillary/fiber ratio in the diaphragm. An increase in diaphragmatic capillary/fiber ratio has been described after 3 weeks of chronic hypoxia [33], however, indirect indicators of tissue hypoxia were not observed in our study. Given changes in blood flow provide a powerful stimulus for angiogenesis [34], [35], it is possible that the severely diminished blood flow to the diaphragm with prolonged MV [3] led to the diminished VEGF mRNA expression (Figure 2), which may result in a capillary rarefaction over time. Although no changes in capillarity were observed in the current study, the time frame (i.e., 24 h) may not have been sufficient to result in structural modifications of the microvasculature. Furthermore, our staining protocol allows quantification of capillaries but cannot discriminate between those that supported red blood cell flow (functional) and those that may not have been be perfused due to the reduced diaphragm blood flow [3]. Future studies using intravital microscopy [36] or pharmacological staining of functional capillaries are needed to address this question. Surprisingly, there was a slight reduction in capillary/fiber ratio in mixed gastrocnemius muscle. Given this muscle is comprised of a mosaic of high and low oxidative fibers [37], this may reflect slightly different anatomical locations of the harvested tissue versus a direct modulation from the protocol. Regardless, this is interesting in that the possibility exists for a rapid rarefaction induced by disuse.

### Limitations of the study

Previous work in VIDD has used sodium pentobarbital as the anesthetic agent because it does not influence specific force production or atrophy in the diaphragm [15], nor does it affect skeletal muscle blood flow [3] in response to prolonged MV. For example, Davis et al. reported no decrease in blood flow to the gastrocnemius over timewhile using sodium pentobarbital as the anesthetic agent [3]. Furthermore, in a variety of cells, barbiturate exposure induces a down-regulation of HIF-1α [38], [39], versus an up-regulation as observed herein. Control animals used in the current study were not temporally aligned with respect to anesthetic duration versus those of the experimental group. Consequently, cause and effect cannot be directly determined. However, spontaneously breathing animals, subjected to the same anesthesia as used in the present study do not display any changes in diaphragm blood flow or oxygenation over time, nor the ability to augment diaphragm blood flow with contractions [3], which suggests that prolonged anesthetics do not alter vascular function nor induce the changes in angio-neogenetic factors described in the current study. However, this possibility cannot be excluded and this should be taken into account when interpreting this data.

## Conclusion

Mechanical ventilation and the associated muscle disuse of both the diaphragm and gastrocnemius muscles induce a change in mRNA expression of angio-neogenetic factors. The observed alterations in angio-neogenetic factors may change vessel architecture if the duration is long enough. Although we did not see structural changes in the current study in the diaphragm with 24 h of MV, there were significant changes in the mRNA of signaling mechanisms that may result in capillary rarefaction in time. The underlying mechanisms may be complex and a result of four possible converging pathways: disuse, inflammation, oxygenation and alterations in blood flow. Future studies are needed to discriminate how these pathways can be manipulated to combat VIDD in patients to facilitate operational care.

## Supporting Information

Figure S1 **Hif1α and GLUT 4 expression in Gastrocnemius tissue.** Hif1α protein levels (left) and GLUT 4 protein levels (right) in Gastrocnemius tissue. There is no change after 24 h of disuse (n = 7 for MV and n = 6 for control). Exposition times: A HIF 10 min, GAPDH 2 sec; B GLUT 4: 5 min, GAPDH 5 sec; MV = Mechanical ventilation.
(TIF)

Figure S2 **Proof of sufficient antibody binding.** Western blots of homogenized HEY cells pre-incubated for 96 h in an hypoxic chamber in comparison to diaphragm tissue (A–C) and gastrocnemius tissue (D)(MV/control). Clear HIF-1α (A), GLUT 4 (B), Hif 2α (C) bands compared to diaphragm tissue and HIF-2α in gastrocnemius tissue (D) in positive controls define proper antibody binding. GA =  Gastrocnemius, Dia = Diaphragm, Exposition times: A Hif 1α 10 min; B GLUT 4 3 min; C HIF 2α 2 min; D HIF 2α 30 sec.
(TIF)

Figure S3 **Glut 1 staining of diaphragm fibers.** Quantification of five images (Area of each 400 µm×300 µm), (A) Nuclei staining (top), Glut 1 staining of muscle fibers (middle) and merged picture (bottom) Scale bar  = 50 µm (B) Fluorescent intensity, #  =  not significant, A.U.  =  aubitrary units (C) Magnification of GLUT 1 staining showing expression of GLUT 1 in cell membrane. Scale bar  = 10 µm Exposition times: Hoechst 48 ms, Rhodamine red: 151 ms. MV  =  mechanical ventilation, Con  =  Control.
(TIF)

Figure S4 **Glut 1 staining of gastrocnemius fibers.** Quantification of five images (Area of each 400 µm×300 µm), (A) Nuclei staining (top), Glut 1 staining of muscle fibers (middle) and merged picture (bottom) Scale bar  = 50 µm (B) Fluorescent intensity, #  =  not significant, A.U.  =  aubitrary units (C) Magnification of GLUT 1 staining showing expression of GLUT 1 in cell membrane. Scale bar  = 10 µm Exposition times: Hoechst 17 ms, Rhodamine red: 69 ms. MV  =  mechanical ventilation, Con  =  Control.
(TIF)

Figure S5 **Protein levels of inhibitory Protein κ B α protein.** Changes in inhibitory Protein κ B α (IκBα)in diaphragm (A) and gastrocnemius tissue (B), with representative western blots. Exposition times: IκBα Diaphragma 4 Min/GAPDH 6 sec..; IκBα Gastroc 1Min 30 sec/GAPDH 7 sec. MV  =  mechanical ventilation, Con  =  Control.
(TIF)
