# Supplementary figures and images for: Prolonged Mechanical Ventilation Alters the Expression Pattern of Angio-neogenetic Factors in a Pre-Clinical Rat Model
Source: PLoS One. 2013 Aug 8;8(8):e70524. doi: 10.1371/journal.pone.0070524 (PMC3738548; doi:10.1371/journal.pone.0070524)

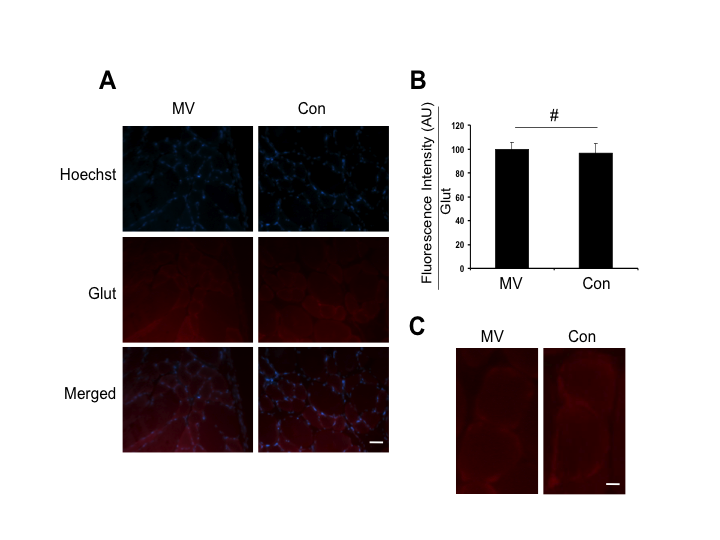

Supplement: Figure S3 — Glut 1 staining of diaphragm fibers. Quantification of five images (Area of each 400 µm×300 µm), (A) Nuclei staining (top), Glut 1 staining of muscle fibers (middle) and merged picture (bottom) Scale bar = 50 µm (B) Fluorescent intensity, # = not significant, A.U. = aubitrary units (C) Magnification of GLUT 1 staining showing expression of GLUT 1 in cell membrane. Scale bar = 10 µm Exposition times: Hoechst 48 ms, Rhodamine red: 151 ms. MV = mechanical ventilation, Con = Control. (TIF) [file pone.0070524.s003.tif]

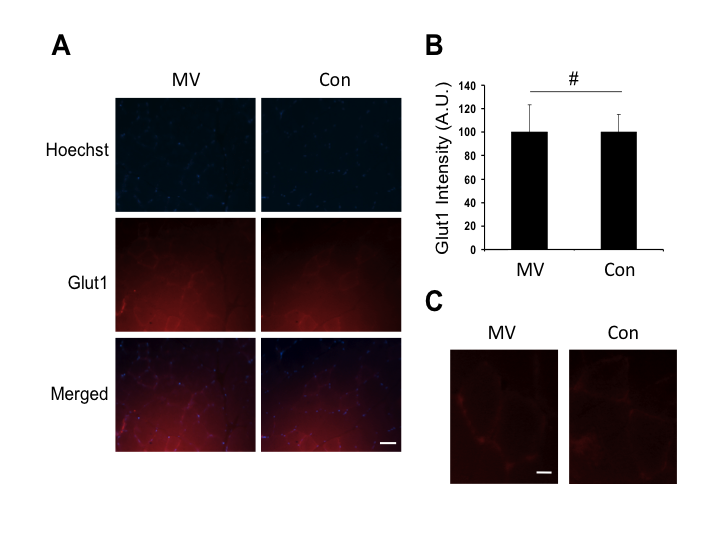

Supplement: Figure S4 — Glut 1 staining of gastrocnemius fibers. Quantification of five images (Area of each 400 µm×300 µm), (A) Nuclei staining (top), Glut 1 staining of muscle fibers (middle) and merged picture (bottom) Scale bar = 50 µm (B) Fluorescent intensity, # = not significant, A.U. = aubitrary units (C) Magnification of GLUT 1 staining showing expression of GLUT 1 in cell membrane. Scale bar = 10 µm Exposition times: Hoechst 17 ms, Rhodamine red: 69 ms. MV = mechanical ventilation, Con = Control. (TIF) [file pone.0070524.s004.tif]

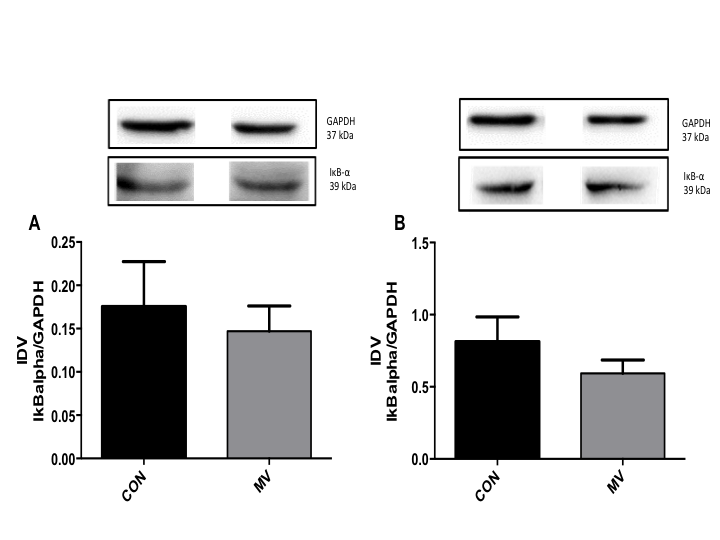

Supplement: Figure S5 — Protein levels of inhibitory Protein κ B α protein. Changes in inhibitory Protein κ B α (IκBα)in diaphragm (A) and gastrocnemius tissue (B), with representative western blots. Exposition times: IκBα Diaphragma 4 Min/GAPDH 6 sec..; IκBα Gastroc 1Min 30 sec/GAPDH 7 sec. MV = mechanical ventilation, Con = Control. (TIF) [file pone.0070524.s005.tif]
